# LAMP3/CD63 Expression in Early and Late Endosomes in Human Vaginal Epithelial Cells Is Associated with Enhancement of HSV-2 Infection

**DOI:** 10.1128/jvi.01553-22

**Published:** 2022-11-09

**Authors:** Aisha Nazli, Ryan Chow, Muhammad Atif Zahoor, Samuel Tekeste Workenhe, Tushar Dhawan, Chris Verschoor, Charu Kaushic

**Affiliations:** a Department of Medicine, McMaster University, Michael G. DeGroote Center for Learning and Discovery, Hamilton, Ontario, Canada; b McMaster Immunology Research Center, McMaster University, Michael G. DeGroote Center for Learning and Discovery, Hamilton, Ontario, Canada; c Department of Pathobiology, Ontario Veterinary College, University of Guelph, Guelph, Ontario, Canada; University of Arizona

**Keywords:** CD63, HSV-2, LAMP3, endosome, epithelial cells, viral replication

## Abstract

Herpes simplex virus 2 (HSV-2) is a lifelong sexually transmitted virus that disproportionately infects women through heterosexual transmission in the vaginal tract. The vaginal epithelium is known to be highly susceptible to HSV-2 infection; however, the cellular mechanism of HSV-2 uptake and replication in vaginal epithelium has not been extensively studied. Previously, we observed that lysosomal-associated membrane protein-3 (LAMP3/CD63) was among the highly upregulated genes during HSV-2 infection of human vaginal epithelial cell line VK2, leading us to posit that LAMP3/CD63 may play a role in HSV-2 infection. Consequently, we generated two gene-altered VK2-derived cell lines, a LAMP3-overexpressed (OE) line and a LAMP3 knockout (KO) line. The wild-type VK2 and the LAMP3 OE and KO cell lines were grown in air-liquid interface (ALI) cultures for 7 days and infected with HSV-2. Twenty-four hours postinfection, LAMP3 OE cells produced and released significantly higher numbers of HSV-2 virions than wild-type VK2 cells, while virus production was greatly attenuated in LAMP3 KO cells, indicating a functional association between LAMP3/CD63 expression and HSV-2 replication. Fluorescence microscopy of HSV-2-infected cells revealed that HSV-2 colocalized with LAMP3 in both early endosomes and lysosomal compartments. In addition, blocking endosomal maturation or late endosomal/lysosomal fusion using specific inhibitors resulted in reduced HSV-2 replication in VK2 cells. Similarly, LAMP3 KO cells exhibited very low viral entry and association with endosomes, while LAMP3 OE cells demonstrated large amounts of virus that colocalized with LAMP3/CD63 in endosomes and lysosomes.

**IMPORTANCE** Collectively, these results showed that HSV-2 is taken up by human vaginal epithelial cells through an endosomal-lysosomal pathway in association with LAMP3, which plays a crucial role in the enhancement of HSV-2 replication. These findings provide the basis for the future design of antiviral agents for prophylactic measures against HSV-2 infection.

## INTRODUCTION

Herpes simplex virus 2 (HSV-2) is a lifelong sexually transmitted infection that targets genital mucosal epithelial cells. The global prevalence of HSV-2 in people between the ages of 16 to 40 exceeded 500 million people in 2016 and is steadily rising (1). Infection is characterized by symptomatic lesions in the genital tract and subclinical shedding following infection. After primary infection, HSV-2 recedes into nuclei of neurons involving, but not limited to, the dorsal root ganglion and spinal cord to establish latency and evade the host’s immune system (2, 3). Intermittently, HSV-2 gets reactivated and can result in genital ulcerative lesions. Currently, there are no effective prophylactics, vaccines, or therapies that confer protection from this virus. HSV-2 is also strongly associated with increased susceptibility to human immunodeficiency virus (HIV) infection (4). HSV-2 is transmitted through physical contact, infecting epithelial cells at mucosal surfaces. Epithelial cells lining the female genital tract are the primary portal of entry of HSV-2 in women via heterosexual transmission. Therefore, this study utilizes immortalized vaginal epithelial cells VK2/E6E7 (VK2). Our lab optimized an air-liquid interface (ALI) culture system to grow VK2 cells, which promotes multilayer structures, mimicking the natural stratified squamous epithelial cells in the vagina (5).

HSV-2 entry into the host cell is a multistep process and involves multiple interactions between viral and host proteins, including proteins on the host cell that act as viral receptors, proteins that assist in viral uncoating and release of the viral genome, as well as those that initiate or aid in viral replication (6). Understanding the mechanism of viral entry and replication is of high interest, as it can lead to the development of prophylactic or therapeutic compounds that stop the initial viral entry or inhibit cell-to-cell spread. Viral uncoating may occur on the cell surface by fusion into the cell membrane or in intracellular compartments such as endosomes (7), but the selection of these pathways is dependent on the target cell type (8). HSV-2 virions utilize five different viral glycoproteins, including glycoprotein B (gB), gC, gD, gH, and gL, for entry into target cells (9, 10). There are various cell receptors utilized for herpesvirus entry, including nectin-1 or -2, herpesvirus entry mediator (HVEM), 3-*O* sulfated heparan sulfate (3-OHS) (10, 11), myelin-associated glycoprotein (MAG), and paired immunoglobulin-like receptor (PILRα) (12). Various epithelial cell types express these receptors, but at this time, it is uncertain which receptors and which pathway of entry predominates for vaginal epithelial cells (8).

In addition to the receptor-mediated pathways, herpesviruses have been also shown to enter cells through an endosomal pathway in a pH-dependent or independent manner (13). In cell types such as Vero, HSV-1 has been shown to penetrate the plasma membrane and deliver its capsid directly into the cytosol. Microtubules then propel the naked capsids to the nuclear region (14–16). However, in other common cell lines, endocytosis of virions is required to initiate successful entry (17). In Chinese hamster ovary (CHO) cells engineered to express entry receptors, such as HVEM, nectin-1, or nectin-2 that bind the HSV gD, entry, as measured by virus-induced gene expression, is inhibited by agents that block either uptake from the cell surface or endosomal acidification (17). HSV-1 uptake by endocytosis can be defined as the delivery of intact, enveloped particles from the cell surface to intracellular vesicles. Whether the endocytic pathway is used by HSV-2 for infecting vaginal epithelial cells is not known.

Lysosomal-associated membrane protein-3 (LAMP3) is also known as CD63/CD208/DC-LAMP and is part of the tetraspanin superfamily, a group of transmembrane proteins found in multicellular eukaryotes (18). These proteins are characterized by their intracellular N and C termini, four transmembrane domains, and two extracellular domains (18). LAMP3 is highly expressed in the lysosomes of mature dendritic cells (19). Lysosomes are membrane-bound organelles involved with endo- and exocytosis, intracellular trafficking, and autophagy (20). They have a major role in the digestion, breakdown, and excretion of host proteins, viral proteins, and many other macromolecules. The function of LAMP3 is not fully elucidated, but it has been shown to be vital to the secretory pathway and cycles between endo- and exocytic compartments in human endothelial cells (18). Other lysosome-associated membrane proteins include LAMP1 and LAMP2, which have been studied more in depth and account for half of the total lysosomal membrane proteins (21). LAMP3 shares 30% and 28% amino acid sequence identity with LAMP1 and LAMP2, respectively, which are constitutively expressed in many more eukaryotic cell types (22).

LAMP3 has been linked to Lujo virus (LUJV) entry in HAP1 cells, where, following viral entry via primary entry receptors, it is engaged with the virus in early endosomes (23). LAMP3 is also involved in influenza A virus replication in A549 human lung epithelial cells, Epstein-Barr exosomal packaging in human embryonic kidney (HEK) 293T cells, and the proliferation of Salmonella enterica serovar Typhimurium (24–26). Tetraspanins, including LAMP3/CD63, are key players in HIV entry and release and also play critical roles at different steps in the viral infection cycle (27). The presence of LAMP3 on the cell surface can regulate host receptor expression. For example, LAMP3 decreases coreceptor CXCR4 expression on plasma membranes of T cells, thus affecting HIV-1 ×4 strain viral entry into T cells (28–30). In addition, LAMP3 increases viral entry of the HIV-1 R5 strain into macrophages, suggesting that LAMP3 specifically controls viral entry facilitated by the coreceptor CCR5 (28–30). Different stages of the viral life cycle are also affected by LAMP3, including reverse transcription and integration into the host genome (31). HIV-1 viral release is facilitated through microdomains enriched in the tetraspanins CD9, CD63, CD81, and CD82 on the plasma membrane in epithelial cells, T lymphocytes, macrophages, and dendritic cells in association with extracellular vesicles and exosomes (32–36). These tetraspanin-rich microdomains are considered a gateway to HIV-1 release (36). LAMP3/CD63, with other tetraspanins, also alters virus-cell fusion and cell-cell spread of infection (37).

Recent gene array data from our lab have shown that when VK2 cells are infected with HSV-2, lysosome-associated membrane protein-3 (LAMP3) is significantly upregulated. Therefore, we decided to investigate the interaction between LAMP3 and HSV-2 infection. We investigated the role of LAMP3 in HSV-2 entry, replication, and shedding in vaginal epithelial VK2 cells. For this purpose, we created two co-cell lines with LAMP3 overexpression (LAMP3 OE) and LAMP3 knockout (LAMP3 KO) on a wild-type vaginal epithelial cell (WT VK2) background. Cell stress and viability assays were conducted to ensure cell viability and growth despite genetic modifications. All three VK2 cell lines, including WT VK2, LAMP3 OE, and LAMP3 KO, were investigated for HSV-2 infection and association of the virus with LAMP3 in endosomal/lysosomal compartments.

## RESULTS

### HSV-2 infection upregulates LAMP3 gene expression in vaginal epithelial cells (VK2).

**FIG 1:**
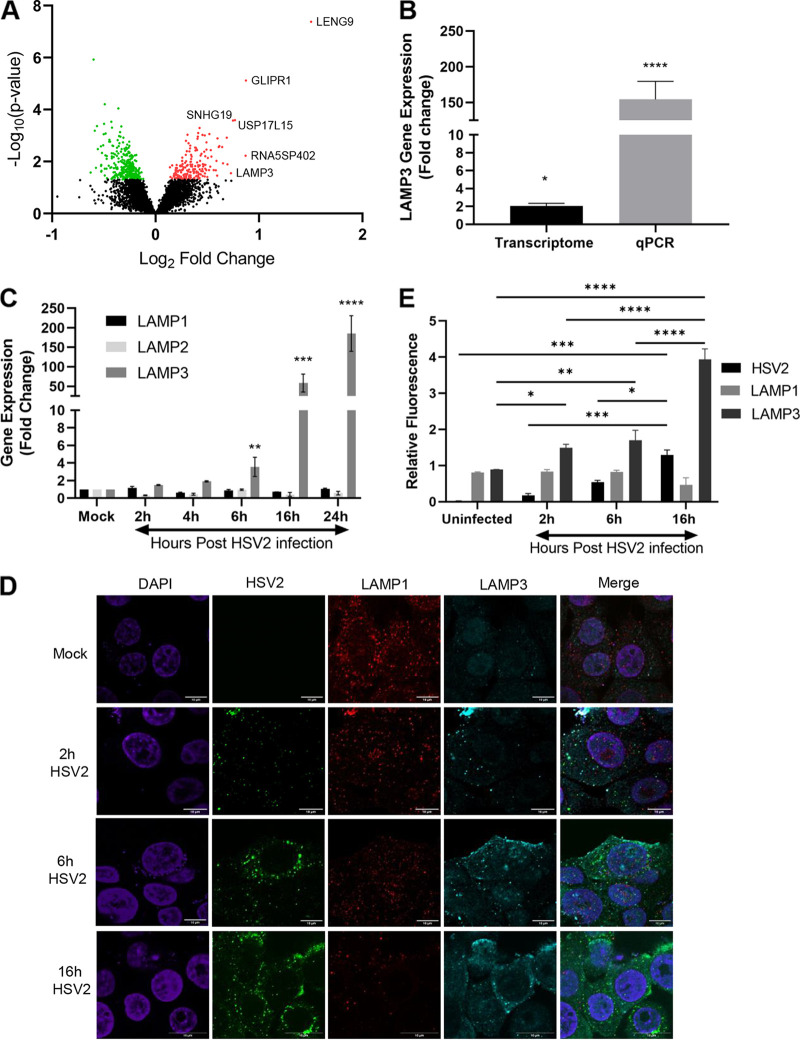
HSV-2 infection upregulates LAMP3 expression in vaginal epithelial cells. VK2 epithelial cells were either uninfected or infected with HSV-2 strain 333 (MOI = 1). (A) After 24 h of infection, RNA was extracted (*n* = 3) and used for transcriptome analysis. Transcriptome data are depicted as a volcano plot with LAMP3 identified as one of 6 highly upregulated (log_2_ fold) genes relative to uninfected VK2 cells. (B) To validate the transcriptome results, qPCR was performed on RNA samples from *n* = 8 experiments. Results from transcriptome analysis and qPCR are shown as fold change in HSV-2-infected (24 h) samples relative to uninfected control. Data are shown as mean ± SEM. Statistical significance is as follows: *, *P* < 0.025, and ****, *P* < 0.0001 by unpaired *t* test. (C) VK2 cells were grown for 7 days in ALI cultures and then infected with HSV-2 strain 333 (MOI = 1), and RNA was extracted at different time points postinfection (*n* = 4) and analyzed for gene expression of LAMP1, LAMP2, and LAMP3. Data are shown as mean ± SEM. Statistical significance is as follows: **, *P* < 0.001; ***, *P* = 0.0003; and ****, *P* < 0.0001 with comparison expression of LAMP3 genes to uninfected basal levels by two-way ANOVA. (D) VK2 cells were infected with HSV-2, and cells were fixed at different time points postinfection. Cells were stained for LAMP3 protein (Cy5, teal), LAMP1 protein (red), and HSV-2 (green), cell nuclei were stained (DAPI, purple) at 0 (mock, uninfected), 2, 6, and 16 h post-HSV-2 infection, and images were captured by confocal microscopy. Representative images are shown. The merged images show all four stains merged. Scale bars, 10 μm. (E) Quantification of fluorescence intensity was measured for HSV-2, LAMP1, and LAMP3 and depicted in the graph. Data are shown as mean ± SEM. Statistical significance is as follows: *, *P* < 0.01; **, *P* < 0.001; ***, *P* = 0.0002; and ****, *P* < 0.0001.

As part of our ongoing research to examine host-pathogen interactions in the female genital tract, we have examined global changes in gene expression of vaginal cell lines during infection with HSV-2. Transcriptional profiling was performed by gene arrays conducted to measure the impact of HSV-2 on host gene expression in VK2 cells of human vaginal origin. VK2 cells were grown in an ALI culture for 7 days and were either left uninfected or infected with HSV-2 333 strain for 24 h as described in Materials and Methods. RNA was extracted and processed for microarray analysis using the human genome ST 2.0 array (Affymetrix) that interrogates 40,716 RefSeq transcripts from 24,838 human genes. Gene expression data were obtained with R software as previously described (38, 39). As identified by microarray analysis, LAMP3 was among the top 6 upregulated genes following HSV-2 infection, as shown in the volcano plot in Fig. 1A. We confirmed this initial observation by performing quantitative PCR (qPCR) validation of LAMP3 mRNA upregulation 24 h after HSV infection of human vaginal VK2 cells (Fig. 1B). While transcriptome analysis indicated a significant 2-fold increase in LAMP3 expression, the qPCR result indicated a highly significant 155-fold increase (*P* < 0.0001).

Having verified the initial transcriptome analysis observation, we next determined the time course of both the LAMP3 mRNA and protein expression in VK2 cells following HSV-2 infection. In addition, we examined the expression of the related genes LAMP1 and LAMP2 to determine if there was any change in their expression. VK2 cells were seeded in ALI cultures for 7 days and infected with HSV-2. RNA was extracted from uninfected (mock) VK2 cells and after 2, 4, 6, 12, and 24 h post-HSV-2 infection of VK2 cells, and expression of all three genes was determined at all time points post-HSV-2 infection by quantitative reverse transcriptase PCR (RT-PCR) (Fig. 1C). The results indicated that LAMP3 is significantly upregulated after HSV2 infection, beginning approximately 6 h after the start of infection, while there was no difference in gene expression of LAMP1 and LAMP2. In addition, LAMP3 and LAMP1 protein expression were compared by fluorescence confocal microscopy (Fig. 1D) and their relative fluorescence quantified (Fig. 1E). Results showed significant upregulation of LAMP3 protein, but not LAMP1 protein, between 6 and 16 h postinfection (Fig. 1D and E). These observations indicate that HSV-2 infection upregulates LAMP3 expression starting early (6 h) in the course of infection.

### HSV-2 infection and replication in VK2 cells are enhanced significantly by LAMP3.

**FIG 2:**
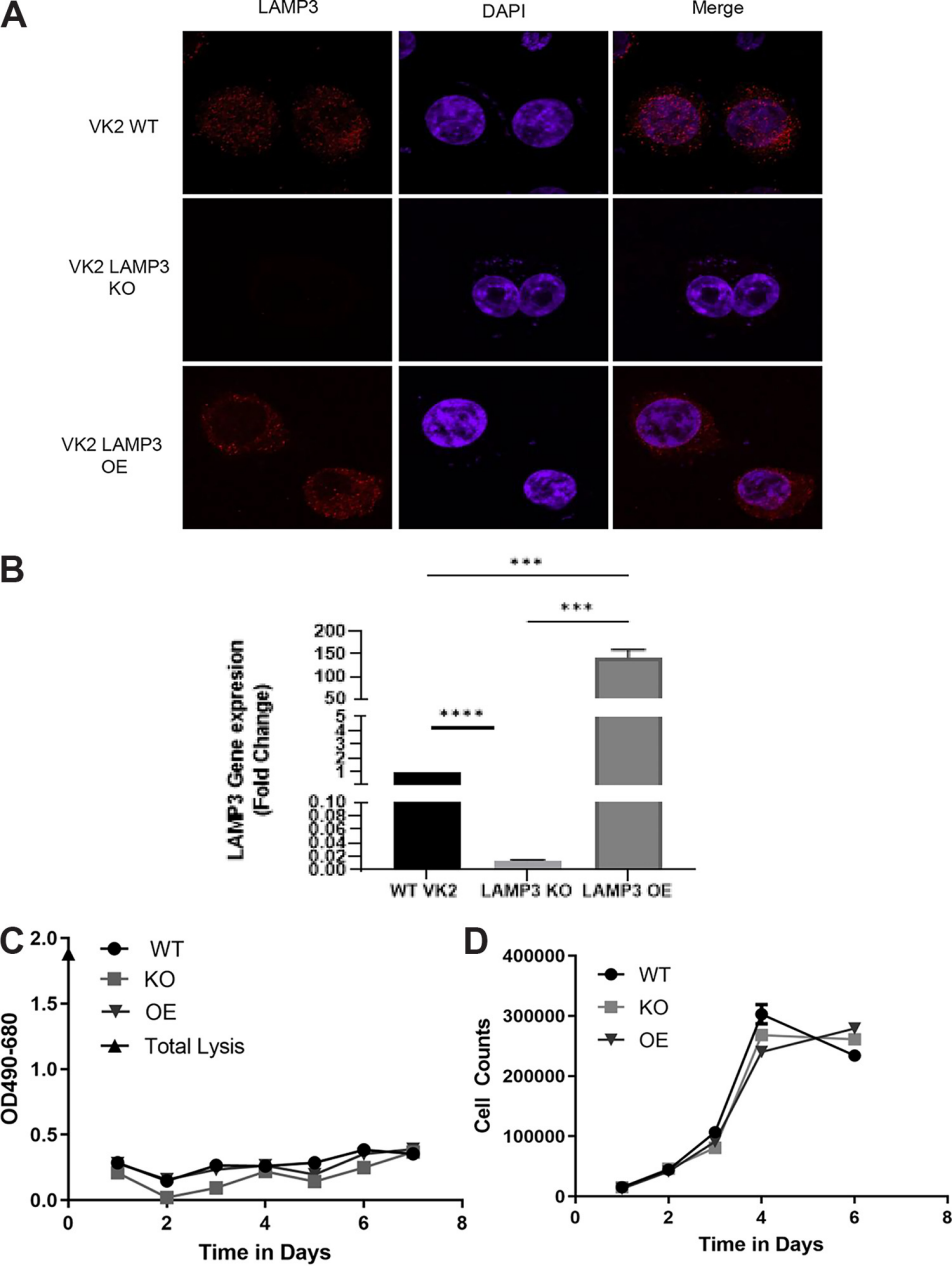
Validation of LAMP3 knockout and overexpression in stable cell lines. (A) Wild-type (WT) VK2 and LAMP3 overexpression (OE) and LAMP3 knockout (KO) cell lines were cultured on chamber slides, fixed, and stained for LAMP3 (red) and DAPI nuclear stain (purple). Confocal images show LAMP3 expression in WT-VK2 and LAMP3 OE but not in KO cell lines. Magnification scale bars, 10 μm. (B) RNA was extracted from WT, LAMP3 OE, and LAMP3 KO cell lines and subjected to qPCR. Data (*n* = 3) are shown as mean ± SEM. Statistical significance is as follows: ***, *P* = 0.0001, and ****, *P* < 0.0001. (C) Supernatants were collected for 7 days from both WT and OE cell lines and subjected to LDH assay. No significant differences were observed between WT and LAMP3 OE or KO cell lines. Data are shown as mean ± SEM (*n* = 3). (D) Trypan blue exclusion test was utilized to determine live cell counts for 6 days. No significant differences in live cell counts were observed in the LAMP3 OE and KO cell lines compared to WT cells. Data shown as mean ± SEM (*n* = 3).

LAMP3 is known to be localized to endosomes and may have a role in secretory pathways (18). Because herpesviruses, including HSV-1, are known to utilize endocytic pathways to enter epithelial cells, we hypothesized that LAMP3 may play a role in the entry and replication of HSV-2 in VK2 cells. To test this hypothesis, we generated two transgenic cell lines from VK2 cells. In the first, LAMP3 overexpression was conferred by a plasmid using a second-generation lentiviral method (40), and in the second, a LAMP3 knockout was generated using the CRISPR/Cas9 methodology (41). Both confocal microscopy and qPCR analyses for expression of LAMP3 confirmed the increased LAMP3 expression in the LAMP3 OE cells and the absence of LAMP3 in the LAMP3 KO cells (Fig. 2A and B). In addition, neither the overexpression nor knockout of LAMP3 affected the viability or growth kinetics of the newly generated cell lines (Fig. 2C and D).

**FIG 3:**
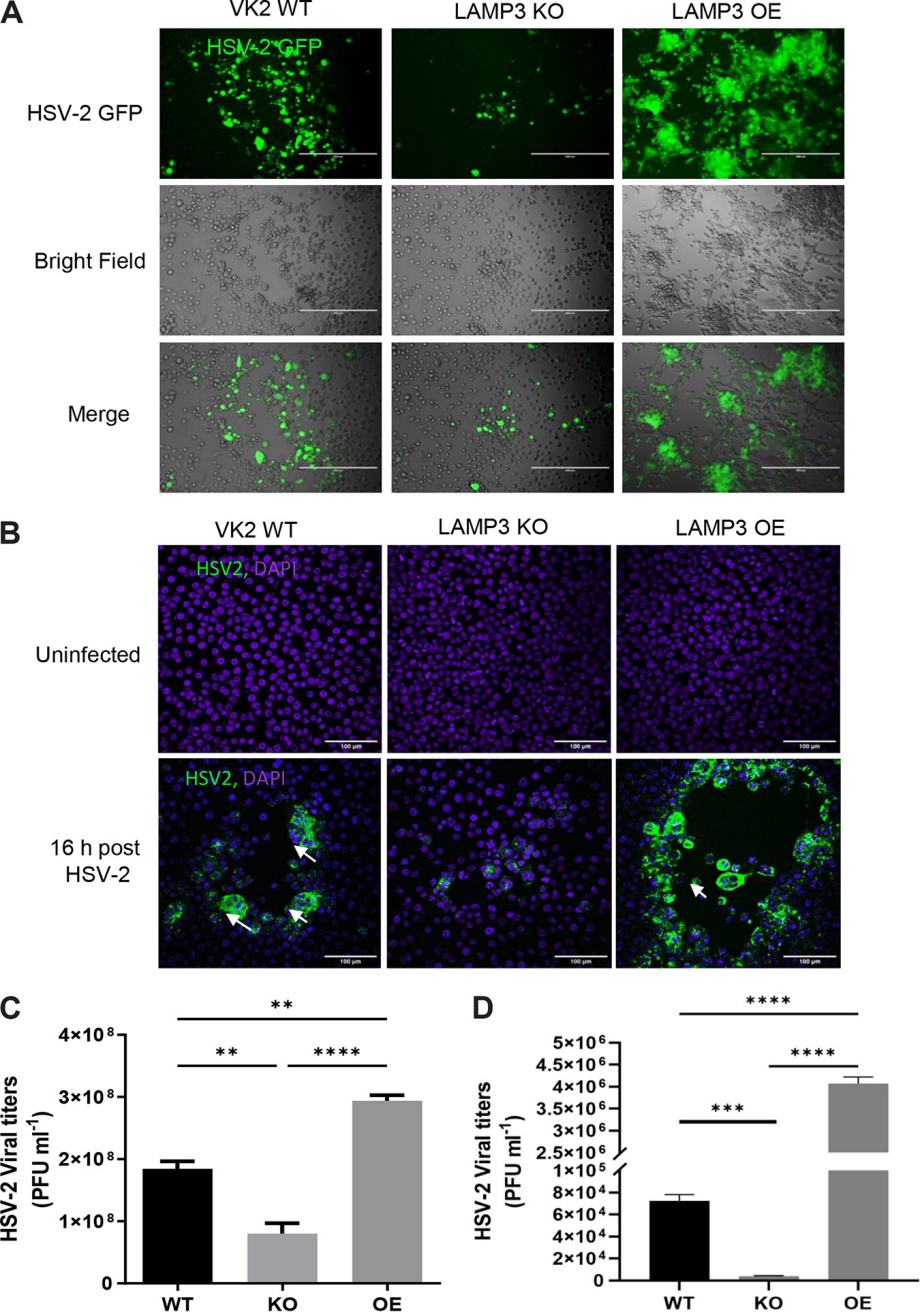
LAMP3 gene expression associated with increased HSV-2 infection. (A) VK2 WT, LAMP3 KO, and LAMP3 OE cells were grown in Transwells and infected with HSV-2 GFP for 16 h. Infected Transwell cultures were fixed and visualized under fluorescence microscope (Evos). Images were captured, and representative images are shown. Magnification bar, 400 μm. (B) VK2 cells in Transwell cultures were also infected with HSV-2 GFP and stained with DAPI after fixation. Images were captured on a confocal microscope with 20× objective. Representative images are shown. Arrows indicate large plaques with syncytium. Magnification bar, 100 μm. (C) Titers of HSV-2 in supernatants taken 24 h post-HSV-2 infection of WT VK2, LAMP3 KO, and LAMP3 OE cells. Data are shown as mean ± SEM. Statistical significance is as follows: **, *P* < 0.005, and ****, *P* < 0.0001. Data are pooled from 4 separate experiments. (D) Cell-associated HSV-2 titers in WT, LAMP3 KO, and LAMP3 OE cell lines, 12 h post-HSV-2 infection. Data are shown as mean ± SEM. Statistical significance is as follows: ****, *P* < 0.0001, and ***, *P* = 0.0003. Data are representative of *n* = 4 experiments.

Having successfully derived both the LAMP3 KO and LAMP3 OE VK2 cell lines, we tested how changes in LAMP3 expression altered outcomes of HSV-2 infection. The KO and OE VK2 cell lines and the WT VK2 cells were grown individually in ALI cultures for 7 days and infected (multiplicity of infection [MOI] of 1) with HSV-2-green fluorescent protein (GFP). Sixteen hours postinfection, the cells were fixed, and images were captured by fluorescence microscopy using an inverted fluorescence microscope (Evos). Infected cells appeared bright green due to the presence of HSV-2 GFP, and the cytopathic effects of HSV-2 were visible, seen by rounding and clustering of cells in the phase-contrast image, formation of plaques, and presence of giant multinucleated cells forming syncytium, compared to uninfected VK2 cells (Fig. 3A). The LAMP3 OE VK2 showed a higher frequency of infected cells with very high levels of virus relative to WT control cells, with very few KO cells containing fluorescent virus. LAMP3 OE cells also showed larger plaque sizes and more infected cells, much more than WT controls and KO cells, as seen in the DAPI (4′,6-diamidino-2-phenylindole)-stained images (Fig. 3B). The near absence of HSV fluorescence in LAMP3 KO cells indicates that the absence of LAMP3 greatly impedes HSV-2 infection of VK2 cells.

To determine the effect of LAMP3 on viral production, the three cell lines were infected with HSV-2, and 24 h postinfection, supernatants were collected, and viral titers were performed using the Vero cell plaque assay (5). LAMP3 gene overexpression significantly increased HSV-2 released into the supernatants compared to WT VK2 cells. Significantly smaller amounts of virus were found in supernatants from infected LAMP3 KO cells than in WT or LAMP3 OE cells (Fig. 3C). To determine differences in intracellular accumulation of HSV-2, all three cell lines were infected with HSV-2 (MOI of 1), and 12 h after infection, noninternalized virus was removed by a brief treatment with trypsin followed by washing. Cells were then subjected to freeze-thaw cycles to release intracellular virus. Viral titers performed on the freeze-thaw samples indicated ~100-fold-larger amounts of intracellular virus in LAMP3 OE cells than in WT, and LAMP3 KO had a smaller amount than WT (Fig. 3D). The intracellular viral titers confirmed the visual differences seen in HSV-2 GFP imaging and indicated that increased expression of LAMP3 enhanced infection and replication of HSV-2 in VK2 cells.

### Intracellular HSV-2 is enhanced by overexpression of LAMP3 in VK2.

**FIG 4:**
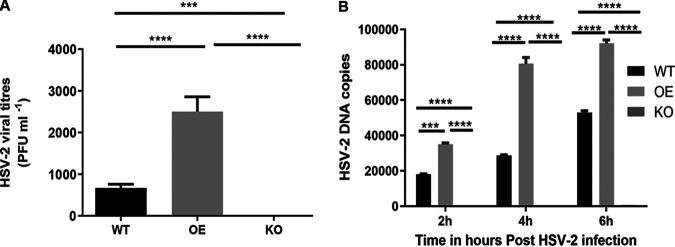
Early intracellular accumulation of HSV-2 is enhanced by overexpression of LAMP3. (A) The WT VK2, LAMP3 OE, and LAMP3 KO cell lines were infected with HSV-2 (MOI of 100), and cell-associated viral titers were assessed in all three cell types 2 h postinfection. Data shown as mean ± SEM are from 1 experiment representative of *n* = 4, each experiment included 3–4 technical replicates. Statistical significance is as follows: ****, *P* < 0.0001. (B) HSV-2 viral DNA was quantified by qPCR using total DNA collected from WT VK2, LAMP3 OE, and LAMP3 KO cells at 2, 4, and 6 h post-HSV-2 infection. Data shown as mean ± SEM from 1 experiment representative of 3 with *n* = 3 to 4 replicates. Statistical significance is as follows: ****, *P* < 0.0001.

Since our results showed that LAMP3 influences overall infection and replication of HSV-2, we decided to examine the different stages of viral infection and replication in more detail. To assess if LAMP3 can alter the early phase of infection, we infected WT VK2, LAMP3 OE, and LAMP3 KO cell lines with HSV-2 (MOI of 100). The high MOI was used to ensure clear observation of HSV-2 uptake in the early phase of infection, when only low numbers of virus would be taken up with typical use of low MOI (MOI of 1) (see 2-h image, Fig. 1D). At 2 h postinfection, the non-cell-associated virus was removed by extensive washing of cells, cells were lysed, and cell-associated viral titers were determined by Vero cell plaque assay. Figure 4A shows the titers of virus from the three cell lines at 2 h postinfection. Data show accumulation of cell-associated virus in WT and OE cells, with significantly higher levels (>3-fold) of HSV-2 detected in LAMP3 OE compared to WT. LAMP3 KO cells had almost no detectable virus (Fig. 4A). Thus, LAMP3 significantly enhanced cell-associated HSV-2 since its overexpression led to high titers of HSV-2 associated with the cells, and its absence in KO cells resulted in negligible cell-associated HSV-2.

In addition to viral titers, we also extracted total DNA from the samples of HSV-2-infected cell lines at 2, 4, and 6 h postinfection and performed quantitative PCR using HSV-2-specific primers and compared these to HSV-2 copy number standards. There were significantly higher HSV-2 DNA copies in LAMP3 OE at all three time points examined than in WT VK2 (Fig. 4B). VK2 KO, on the other hand, showed minimal DNA copies at 2 h, and there was no significant change in the low level of viral DNA over time. Thus, intracellular HSV-2 is correlated with expression of LAMP3, demonstrated by a significantly larger amount of viral DNA present in the LAMP3 OE cell line.

### LAMP3 expression is enhanced after HSV-2 infection and associated with increased endocytic uptake of HSV-2.

**FIG 5:**
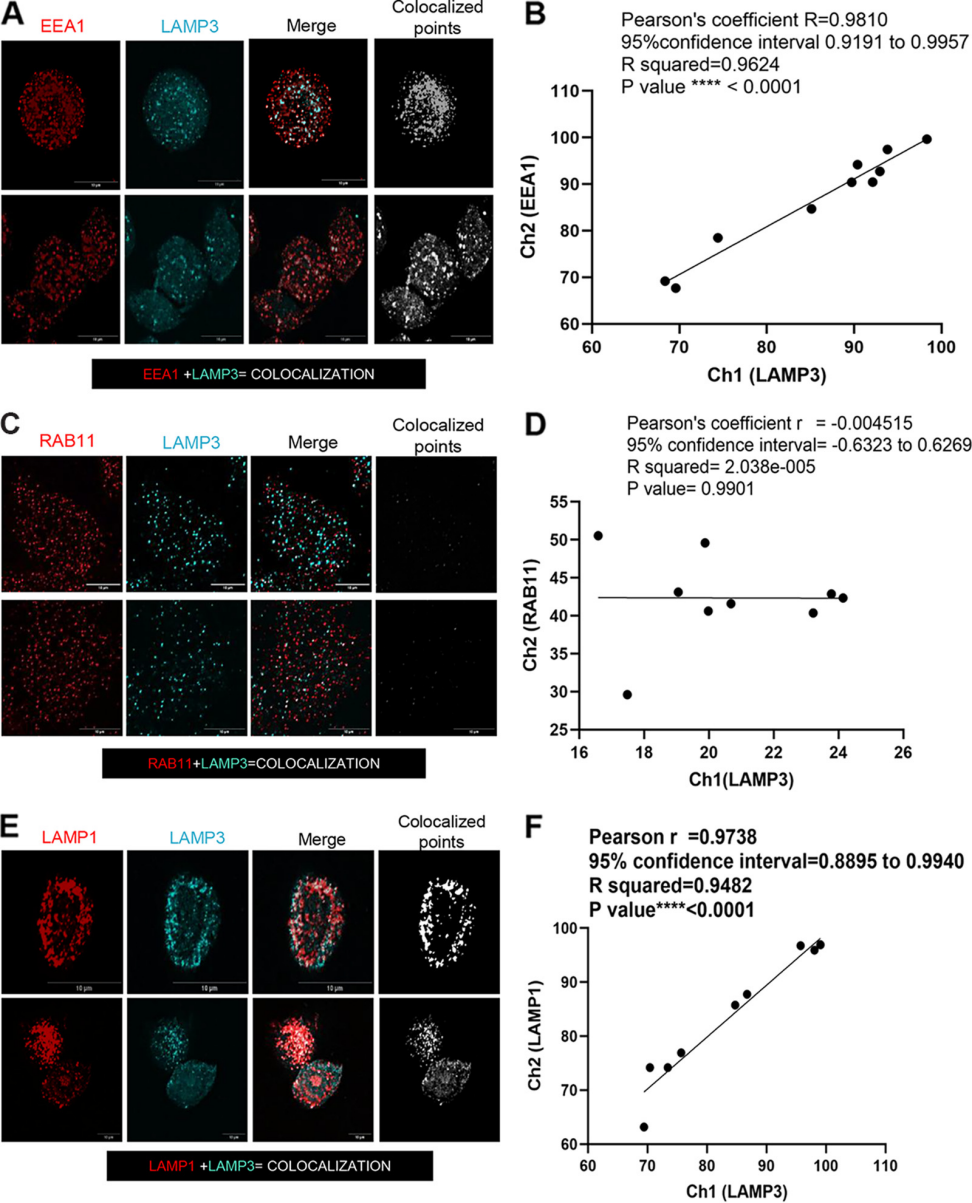
LAMP3 colocalization with EEA1 and LAMP1 in early endosomes. (A) WT VK2 cells were stained for LAMP3 and EEA1. Two sets of representative images (top row, single cell; bottom row, multicell) were captured with a Nikon (Eclipse Ti2) inverted laser confocal microscope. Images show single colors for EEA1 (red), LAMP3 (cyan), and colocalization color (white). Colocalization points were then altered to white in a single image on a black background created by ImageJ software. Colocalized spots were captured by Coloc 2 plugin for ImageJ software. (B) Colocalization between LAMP3-EEA1 was measured (*n* = 10 images) and plotted as described in Materials and Methods. (C) Cells were stained for LAMP3 and RAB11 marker of recycling endosomes, with very little overlap seen. (D) Colocalization between RAB11 and LAMP3 was measured (*n* = 10 images), and correlation was plotted. (E) WT VK2 cells were stained for LAMP1 and LAMP3, and images were captured. Colocalization points between LAMP1 and LAMP3 were assessed and created in ImageJ. (F) Colocalization between LAMP3 and LAMP1 was measured (*n* = 10 images), and the correlation graph was plotted.

LAMP3 has been associated with endosomal pathways (23), and we observed increased HSV-2 uptake with overexpression of LAMP3. We therefore questioned if LAMP3 and internalized HSV-2 could be colocalized within endosomes using the early endosomal marker EEA1. First, WT VK2 cells were stained with fluorescent antibodies against EEA1 and the LAMP3 protein to examine their intracellular localization by confocal microscopy. Images were then analyzed on ImageJ software for colocalization of LAMP3 and EEA1 (Fig. 5A). The results indicated that there was significant colocalization (white) between the two proteins LAMP3 (cyan) and EEA1 (red), indicating the presence of LAMP3 in early endosomes. The high degree of colocalization is evident for LAMP3 and EEA1 in the transformed white-on-black image for all colocalized points (Fig. 5A). The colocalization of LAMP3 and EEA1 was calculated using the colocalization threshold plugin in the ImageJ program, and the correlation of the colocalization between LAMP3 and EEA1 was found to be highly significant (*R*^2^ = 0.96; *P* < 0.0001) (Fig. 5B). In contrast, there was no significant correlation (*R*^2^ = 2.038E-005; *P* = 0.9901) for colocalization of LAMP3 and RAB11, a marker of recycling endosomes (Fig. 5C and D). LAMP1 was also used as a positive control to see the colocalization between both lysosomal-associated proteins, LAMP1 and LAMP3 (Fig. 5E and F). Both lysosomal proteins as expected showed high degree of correlation of colocalization (*R*^2^ = 0.94; *P* < 0.0001).

**FIG 6:**
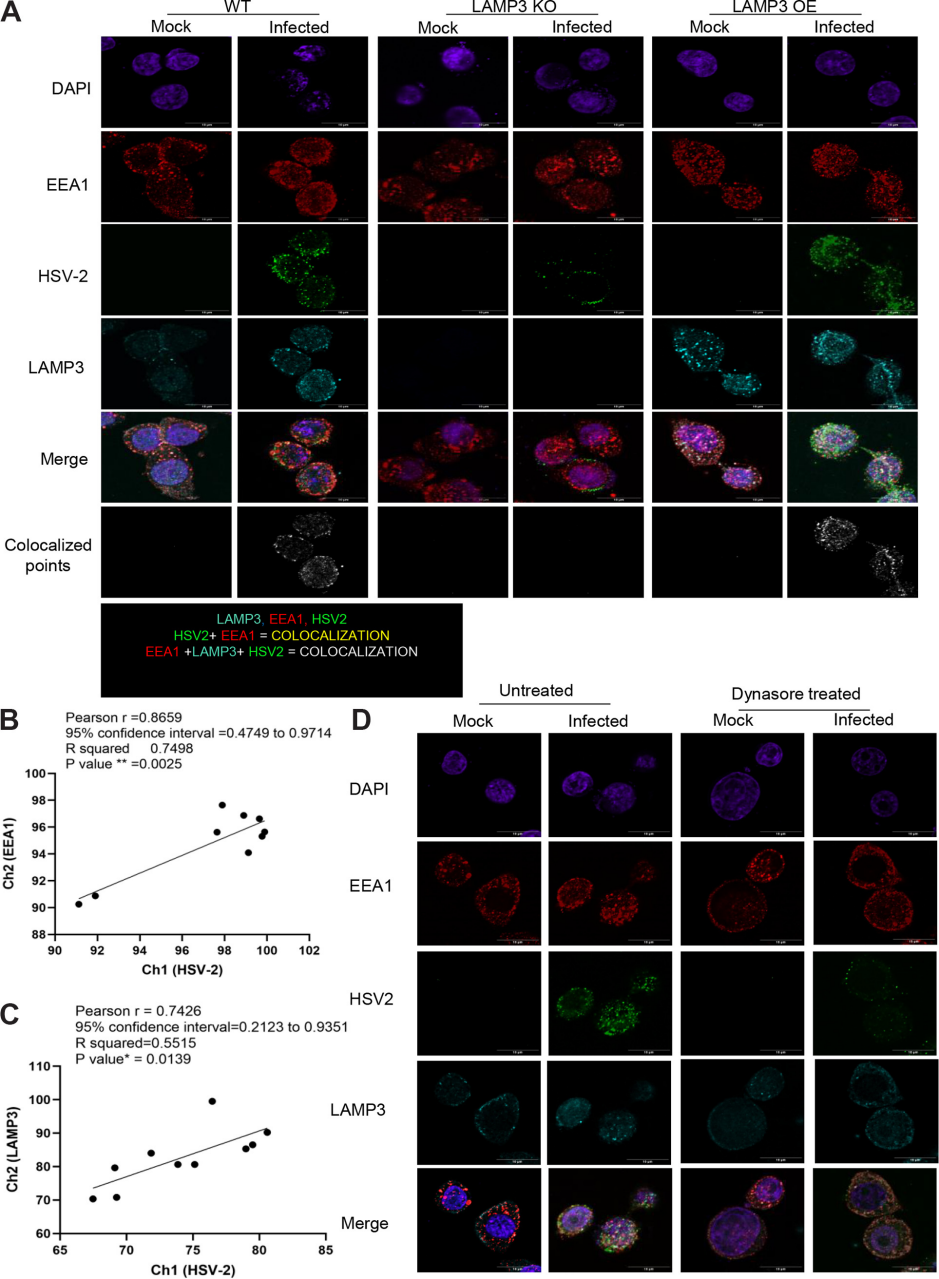
LAMP3 facilitates HSV-2 entry into early endosomes in vaginal epithelial cells. (A) All three cell lines, WT, LAMP3 OE, and LAMP3 KO, were infected with HSV-2 for 1 and 3 h, after which cells were fixed and stained using immunofluorescent antibodies against LAMP3 (teal), EEA1 (red), and HSV-2 (green). Mock indicates images from noninfected control. Individual channel fluorescence and merge images and images showing colocalized spots between LAMP3 and HSV-2 was created using the Coloc 2 plugin in the ImageJ program. (B and C) Colocalized pixels between HSV-2 with EEA1 (B) and HSV-2 with LAMP3 (C) were measured (*n* = 10 images) and shown as correlation graphs, with Pearson values and statistical significance shown. (D) WT VK2 cells were untreated or treated with the endosomal inhibitor Dynasore for 1 h, after which cells were infected with HSV-2. The cells were fixed at 3 h postinfection and then stained with specific antibodies against LAMP3 (blue), EEA1 (red), and HSV-2 (green). Representative images are shown from similar results in 4 to 5 experiments. Images were captured using a fluorescence confocal microscope. Magnification scale bars, 10 μm.

Next, we determined if HSV-2 colocalizes with LAMP3 in EEA1-positive early endosomes. WT VK2, LAMP3 KO, and LAMP3 OE cell lines were grown on chamber slides and infected for HSV-2. At 3 h postinfection, the cells were fixed and stained with fluorescent antibodies against LAMP3, EEA1, and HSV-2. Uninfected cells from respective cell lines were also fixed and stained as controls. WT cells that were not infected showed clusters of EEA1 expression located in the periphery of the cell, most of it colocalized with LAMP3 expression. However, a small amount of diffuse LAMP3 expression was also seen toward the center of cells, but this expression did not show colocalization with EEA1 (Fig. 6A). LAMP3 expression and overlap EEA1 was further increased in OE cells (Fig. 6A), while LAMP3 expression was not seen in the KO cells. After 3 h of HSV-2 infection, LAMP3 KO cells showed normal EEA1 expression, negligible HSV-2 uptake, and no colocalization. In contrast to WT VK2, OE cells showed colocalization clusters of HSV-2, EEA1, and LAMP3. WT VK2 and OE also showed an overlap of EEA1 with LAMP3 at 3 h postinfection and clear colocalization of HSV-2 GFP and LAMP3 proteins (white) at 3 h postinfection. These results indicated that LAMP3 localizes in EEA1-positive early endosomes and HSV-2 colocalizes with some LAMP3 and EEA1 endosomes in the early stages of infection (Fig. 6A). To further evaluate the presence of virus in early endosomes in association with LAMP3, correlation of colocalization quantitation was done between EEA1 and HSV-2 (Fig. 6B) and LAMP3 and HSV-2 (Fig. 6C) fluorescence. Both EEA1 and LAMP3 showed a high correlation with HSV-2 (*R*^2^ = 0.74, *P* < 0.002, and *R*^2^ = 0.55, *P* < 0.01, respectively), indicating again that HSV-2 enters VK2 cells through early endosomes. To validate our observation that HSV-2 uptake was through the endosomal pathway and to further support the association of LAMP3 with HSV-2 in endosomes, we utilized the endosome inhibitor Dynasore, a GTPase inhibitor that rapidly and reversibly inhibits dynamin activity, preventing clathrin-mediated endocytosis (42). WT VK2 cells were seeded into chamber slides and pretreated with medium or Dynasore and infected with HSV-2 and then fixed and stained at 3 h postinfection for EEA1, LAMP3, and HSV-2. Results show that virtually no HSV-2 was observed in Dynasore-treated cells at 3 h; neither was there any increase in expression of LAMP3 or colocalization of EEA1, LAMP3, and HSV-2 (Fig. 6D). Overall, these results show that HSV-2 is internalized predominantly through the endocytic (EEA1) pathway in VK2 cells, that LAMP3 is upregulated by HSV-2 infection, and that LAMP3 enhances uptake of HSV-2 into the early endosomes.

### LAMP3 expression correlates with viral gene expression.

**FIG 7:**
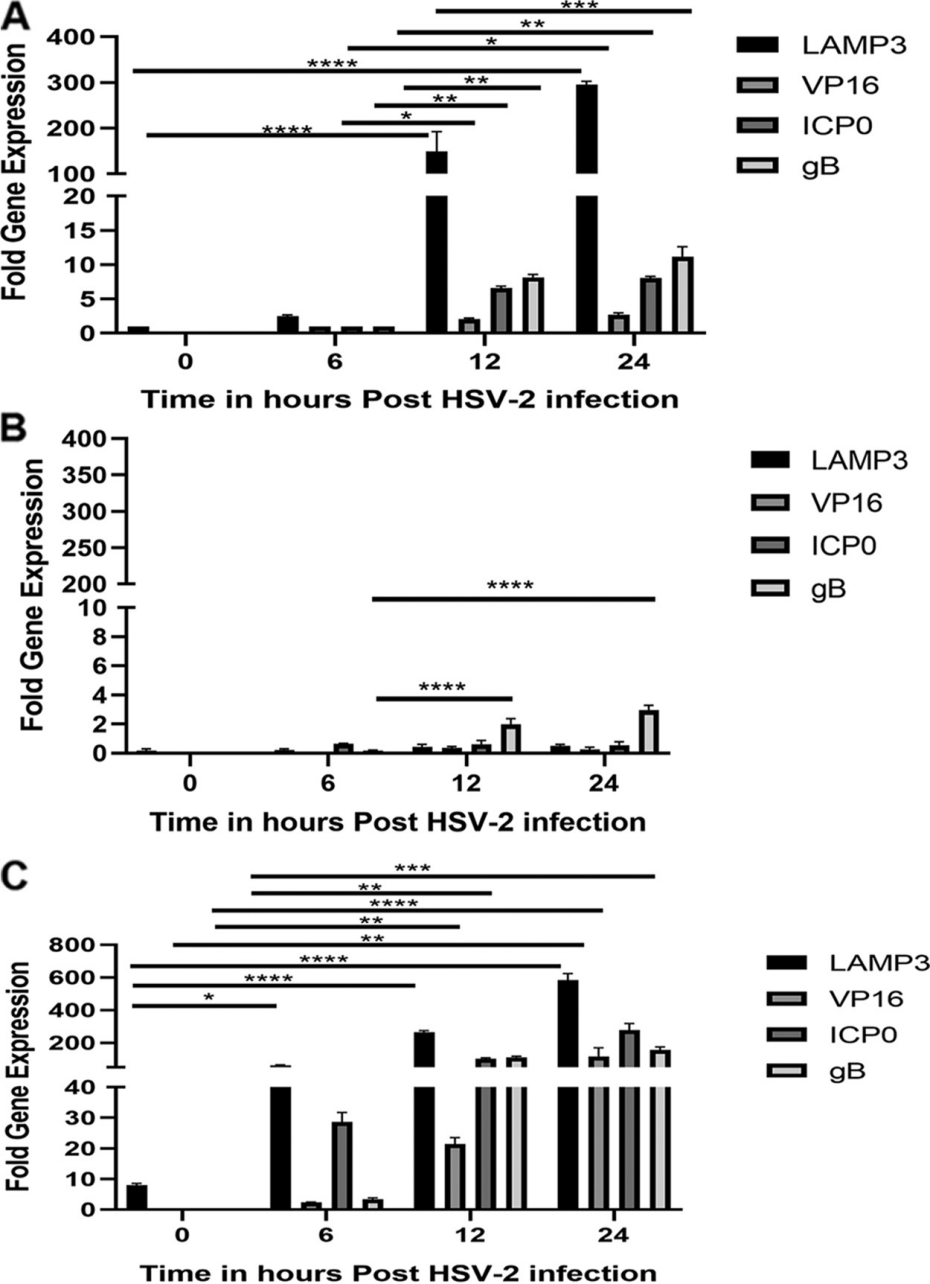
LAMP3 upregulation with HSV-2 infection is coincident with increased viral gene expression. RNA was extracted from WT VK2 (A), LAMP3 KO (B), and LAMP3 OE (C) cells at 0 (uninfected), 6, 12, and 24 h post-HSV-2 infection. HSV-2 gene expression was assessed by qPCR for immediate early gene ICP0 and late genes VP16 and gB using RNA taken at each time point. The fold change in viral gene expression at 12 and 24 h was determined using the 6-h time point in WT VK2 as baseline. LAMP3 gene expression is shown as fold change in using uninfected WT VK2 0 h time point as baseline. Data indicate mean ± SEM fold change for *n* = 3 samples. Statistical significance is as follows: *, *P* < 0.05; **, *P* < 0.001; ***, *P* = 0.0003; and ****, *P* < 0.0001.

The results so far indicated that increasing LAMP3 expression enhanced HSV-2 uptake through the endocytic pathway and in the absence of LAMP3, there was very little uptake of HSV-2. In order to determine how LAMP3 affected postentry replication of HSV-2, we isolated RNA from uninfected WT VK2, KO, and OE cells (time zero) and at 6, 12, and 24 h postinfection and performed reverse transcriptase quantitative PCR (RT-qPCR) for LAMP3, the HSV-2 immediate early gene ICP0, and the late genes VP16 and gB. ICP0 launches the program for viral infection and induces the expression of other essential viral genes and thus is an indicator of the early stage of viral replication (43). The time course study indicated that increased expression of LAMP3 in WT post-HSV-2 infection was coincident with expression of HSV-2 immediate early and late genes at 12 and 24 h postinfection (Fig. 7A). In the absence of LAMP3 (LAMP3 KO; Fig. 7B), viral gene expression was significantly reduced from 10-fold to 2-fold for gB and negligible for ICP0 and VP16. In contrast, in LAMP3 OE cells, LAMP3 expression was upregulated earlier, starting at the 6-h time point, and correlated with much higher upregulation of viral genes (30- to 200-fold compared to 2- to 10-fold in WT cells by 12 h) (Fig. 7C). Thus, LAMP3 expression and upregulation correlated with increases in HSV-2 gene expression, indicating enhanced viral replication by 6 to 12 h postinfection.

### LAMP3 colocalizes with HSV-2 in late endosomes.

**FIG 8:**
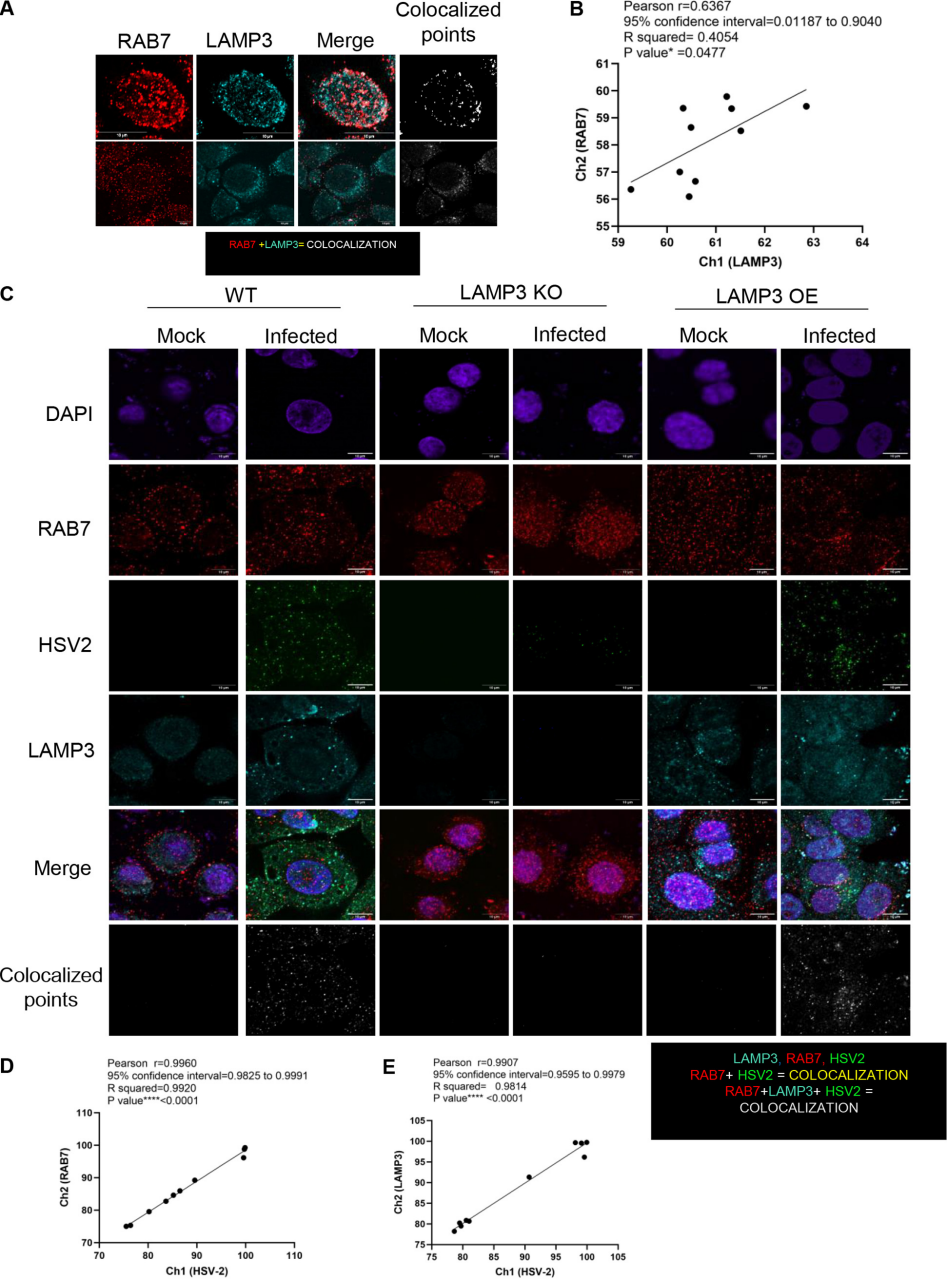
LAMP3 colocalizes with HSV-2 in late endosomes. (A) WT VK2 cells were stained with antibodies against RAB7, a late endosome marker, in addition to LAMP3. Images were captured using a fluorescence microscope. Representative images are shown, with top panels showing single cell at high magnification and bottom panel showing multiple stained cells. The colocalization signal was measured by ImageJ software and is shown as white colocalized points (right). (B) The colocalization was also quantified for RAB7 and LAMP3 in WT cells (*n* = 10 images) and presented as a correlation graph with statistical evaluation. (C) All three cell lines, WT VK2, LAMP3 OE, and LAMP3 KO, were fixed, uninfected (mock), or 8 h after HSV-2 infection, and then stained as in panel A, but with the addition of anti-HSV-2 antibody. Representative images are shown with RAB7, LAMP3, and HSV-2 staining. The bottom images show colocalized points between LAMP3 and HSV-2 after 8 h postinfection. Data are *n* = 3 independent replicates. Magnification scale bars, 10 μm. (D and E) Correlation graphs of colocalized (pixels) between HSV-2 and RAB7 (D) and HSV-2 and LAMP3 (E). Pearson values and statistical significance are shown.

The results thus far indicated the role of LAMP3 in the uptake of HSV-2 into the early endocytic compartment and correlation of LAMP3 expression with viral gene replication. This, combined with the observations that viral shedding correlated with LAMP3, led us to examine LAMP3 and HSV-2 within the late endosome compartments. WT VK2 cells were stained for LAMP3 and late endosomal marker Ras-related protein 7 (RAB7). Initial experiments indicated colocalization of LAMP3 with both RAB7 (Fig. 8A) and LAMP1 (Fig. 5E and F). The colocalization correlation between RAB7 and LAMP3 was found to be statistically significant (*R*^2^ = 0.4, *P* < 0.04) (Fig. 8B), but not as high as between LAMP3 and EEA1.

Next, colocalization of LAMP3 with HSV-2 in late endosomes (RAB7 positive) was evaluated by fluorescence microscopy in WT VK2, LAMP3 KO, and LAMP3 OE cells (Fig. 8C). Cells were infected with HSV-2 or left uninfected and, after 8 h, were stained with antibodies against LAMP3, HSV-2, and RAB7 (Fig. 8C). Quantitative correlation results indicated substantial colocalization of HSV-2 with LAMP3 and RAB7, indicating association with late endosomes (Fig. 8D and E) in HSV-2-infected WT VK2 cells (*R*^2^ = 0.99, *P* < 0.001 for both). The LAMP3 KO cells showed no colocalization of HSV-2 with RAB7 (late endosomes), indicating that LAMP3 was associated with HSV-2 passage through late endosomal compartments. In contrast, LAMP3 OE cells showed very high colocalization signals for HSV-2 with LAMP3 in late endosomes.

### Replication of HSV-2 in vaginal epithelial cells is enhanced by late endosomes and lysosomes.

**FIG 9:**
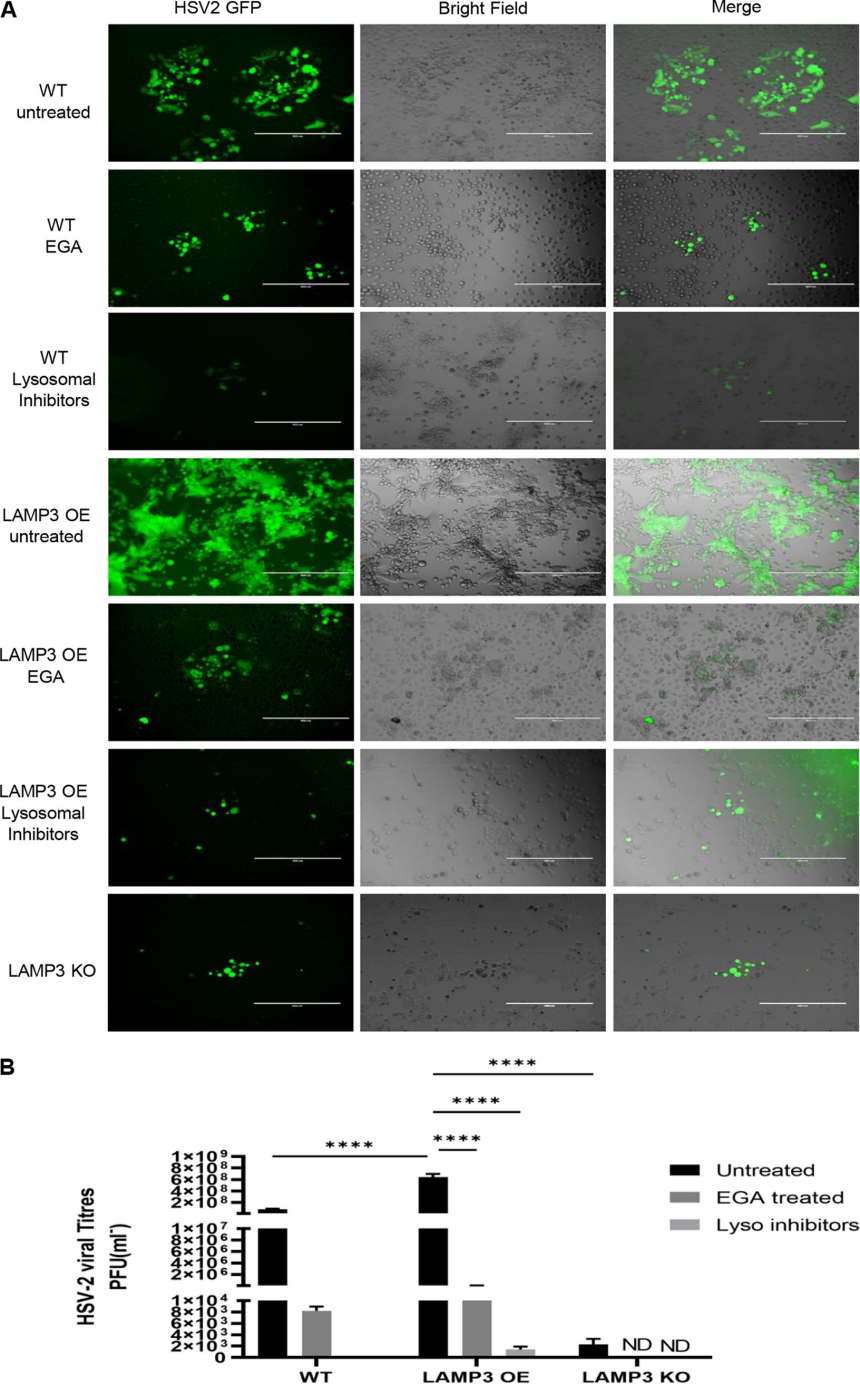
Effect of late endosome and lysosome inhibitors on HSV-2 infection in vaginal epithelial cells. (A) WT VK2 and LAMP3 OE were pretreated with endosomal pathway inhibitor EGA or a cocktail of lysosomal inhibitors and then infected with HSV-2 GFP and cultured for 16 h. Images were captured in EVOS microscope showing green HSV-2 GFP-infected cells. Scale bar, 400 μm. LAMP3 KO cells were also used for comparison after 16 h of HSV-2 infection. (B) HSV-2 viral titers in supernatants collected 24 h postinfection of WT VK2 and LAMP3 OE cells that were pretreated with endosomal or lysosomal inhibitors or not prior to infection with HSV-2. In addition, LAMP3 KO cells were infected with HSV-2 for comparison. Data shown represent mean ± SEM for *n* = 3 experiments each with 3 to 4 replicates. Statistical significance is as follows: ****, *P* < 0.0001.

To test the requirement for functional late endosomes and lysosomes in HSV-2 replication, we used EGA to prevent endosomal trafficking and maturation into acidified late endosomes (44) and a mixture of pepstatin A, leupeptin, and E-64, which are reported to successfully inhibit the lysosomal degradation pathway (45). WT VK2 cells were treated with either EGA or the lysosomal inhibitors and then infected with HSV-2 GFP for 16 h prior to fixation for fluorescence microscopy. Inhibition of late endosome or lysosomal compartments resulted in greatly reduced HSV-2 GFP in VK2 cells (Fig. 9A). In a separate study, cells were treated with late endosome and lysosomal inhibitors prior to HSV-2 infection, and then, culture supernatants were obtained to determine HSV-2 titers as a measure HSV-2 replication. For comparison, similar measures of HSV-2 titers were determined for LAMP3 KO cells. We observed greatly reduced HSV-2 shedding (50-fold lower) compared to untreated control cells when either the late endosomal or lysosomal inhibitors were used (Fig. 9B). The low levels of virus production were similar to LAMP3 KO cells (Fig. 9B). Thus, late endosome and lysosome functions enhance HSV-2 replication, and the absence of LAMP3 inhibits viral replication to a similar magnitude.

## DISCUSSION

HSV-2 has the ability to exploit host cellular responses to support viral replication and to cause lifelong chronic infection. Our previous unpublished data on HSV-2-infected VK2 showed that LAMP3 was one of the most upregulated genes in vaginal epithelial cells after HSV-2 infection. This study was designed to determine if there is any functional role for LAMP3 during HSV-2 infection. Our results indicated that LAMP3 is associated with early and late endosomal and lysosomal localization that can facilitate HSV-2 entry and replication.

Our use of the LAMP3 OE and LAMP3 KO cell lines indicated that HSV-2 infection is altered with changes in the expression of LAMP3. The LAMP3 OE cells showed significantly higher titers of HSV-2 released in the supernatant 24 h postinfection than WT cells. Infection of LAMP3 KO cells resulted in very little release of HSV-2 by 24 h postinfection compared to WT or OE cells. From these data, we conclude that LAMP3 significantly enhances infection and replication of HSV-2 in vaginal epithelial cells. This is consistent with LAMP3/CD63’s role shown in previous literature where knockdown of LAMP3/CD63 impeded VSV-LUJV infection of endothelial cells (23), and LAMP3 knockdown in lung epithelial cells resulted in reduced replication of influenza virus (25).

HSV-2 utilization of early endosomes for viral entry has been examined in the context of HeLa and Chinese hamster ovary cells (17). We demonstrated, by staining and confocal microscopy, the colocalization of HSV-2 and the early endosomal marker EEA1. This indicates that in VK2 vaginal epithelial cells, HSV-2 mainly enters through endocytosis. Furthermore, we show that when LAMP3 is overexpressed, it colocalizes with both HSV-2 and EEA1, and the amount of HSV-2 present in early endosomes is increased relative to WT vaginal epithelial cells. In contrast, when LAMP3 is knocked out, HSV-2 colocalization with EEA1 is not observed, and overall internalization of HSV-2 is greatly reduced. Therefore, LAMP3 appears to play a role in mediating HSV-2 entry via the endocytic pathway in VK2 cells such that increased LAMP3 results in higher numbers of HSV-2 virus in early endosomes. This result is similar to the observations in the influenza virus and lung epithelial model where LAMP3 and influenza A viral proteins were colocalized in early endocytic vesicles (25).

We utilized qPCR to determine if LAMP3 expression and viral gene expression were associated early after infection. ICP0 and VP16 were evaluated as immediate early HSV-2 genes that are expressed right after infection. These immediate early genes code for proteins that start the lytic program and induce the expression of other essential viral genes (43). In the normal VK2 cells, ICP0 and VP16 expression increased coincidently with increased LAMP3 expression. This parallel increased expression profile is consistent with LAMP3 enhancing viral replication, resulting in increased expression of ICP0 and VP16.

There are multiple processes involved in viral uptake, uncoating, and release of the viral genome. Viral uncoating may occur on the cell surface by fusion into the cell membrane or on cellular compartments inside the cells (7). It has been observed that once HSV-2 enters the cells through the endocytic pathway, a low pH is required for fusion of the viral envelope with the endosomal membrane to release viral contents into the cytosol (17). After entry via the endosomal system, there are two possible pathways the HSV-2 virion could use to enter the cytosol. The first one involves the HSV-2 envelope fusing with the early endosomal membrane and releasing its viral contents into the cytoplasm for replication. The other involves HSV-2 remaining in the early endosomes until it acidifies and matures into late endosomes, before being released after fusion with lysosomes (17). Our results indicated that HSV-2 primarily utilizes the latter pathway. HSV-2 mainly stays intact in early endosomes that contain LAMP3 (Fig. 5). When endosomes are acidified, they mature into late endosomes and express RAB7. We stained wild-type cells for RAB7 and LAMP3 and observed that there is a colocalization signal (Fig. 7), which confirmed the previous reports that LAMP3 is a component of late endosomal membranes (18). We utilized EGA, a compound that prevents endosomal trafficking and maturation into acidified late endosomes (44), to determine if HSV-2 replication depended on late endosomal transition and observed that cells treated with EGA had very low levels of HSV-2. These results were consistent with those observed when LAMP3 was knocked out. A similar observation was made using lysosomal functional inhibitors. This indicates that in VK2 cells, HSV-2 may require LAMP3 to proceed to the acidic environment of the late endosome-lysosome compartment prior to membrane fusion and entry into the cytosol. Recent studies have also shown that viruses such as beta-coronavirus, human cytomegalovirus (HCMV), and reovirus can use trafficking through late endosomes and lysosomes as an egress pathway (46–48). Therefore, it is likely that colocalization of HSV-2 with LAMP3 in late endosomes/lysosomes indicates that HSV-2 may also be using this as an egress pathway.

In conclusion, we demonstrate that HSV-2 utilizes the endosomal pathway in VK2 cells for enhancing its replication. It colocalizes with LAMP3 throughout the course of viral infection, from early endosomes to late endosomes and lysosomes, and depletion of LAMP3 significantly reduces HSV-2 entry into VK2 cells and subsequent passage to the intracellular compartments needed for replication. Understanding the preferred pathways for entry and replication in vaginal epithelial cells will assist in designing prophylactic intervention strategies to decrease HSV-2 infections in women.

## MATERIALS AND METHODS

### Cell lines.

The WT VK2 vaginal epithelial cell line (ATCC CRL-2616) was derived from normal human vaginal mucosal tissue and immortalized using a retroviral vector. WT VK2, LAMP3 OE, and LAMP3 KO transgenic cell lines were grown and cultured in keratinocyte serum-free medium (KSFM) (Life Technologies, Carlsbad, CA, USA). The KSFM was supplemented with 0.1 ng/mL of human epidermal growth factor, 0.05 mg/mL of bovine pituitary extract (Life Technologies), and 0.4 mM CaCl_2_ and 100 units/mL penicillin-streptomycin antibiotics (Invitrogen, Carlsbad, CA, USA). HEK 293T cells containing the SV40 T-antigen were cultured in Dulbecco’s modified Eagle’s medium with 4.5 g/L of glucose, supplemented with 10% fetal bovine serum (FBS). Vero cells were cultured in minimum essential medium eagle-alpha modification (α-MEM), supplemented with 5% fetal bovine serum of l-glutamine, 100 units/mL penicillin-streptomycin, and HEPES.

### Generation of LAMP3 overexpression and knockout cell lines.

The LAMP3 overexpression vector was constructed by cloning the coding sequence of LAMP3 into the pHR-CMV-ΔN-IκBα-IRES-Puromycin vector (40). The open reading frame LAMP3 gene (GenBank accession number NM_014398) was PCR amplified using primers 5′-CGG CAC AGG TAG GTT TCT CT-3′ and 5′-ATT TCC CAA CAT CCA TCC TG-3′ containing BamHI and EcoRI restriction enzyme sites at each end and cloned into pHR-CMV-ΔN-IκBα-IRES-Puromycin (40), essentially by replacing the ΔN-IκBα region with LAMP3. The resulting plasmid was designated pHR-CMV-LAMP3-IRES-Puromycin.

For CRISPR/Cas9-mediated knockout of the LAMP3 gene, single guide RNAs (sgRNAs) targeting LAMP3 were designed using the CRISPR Design Tool (http://crispr.mit.edu/). The targeting sequence lies in the first exon of the LAMP3 gene (forward, 5′-CAC CGC ATC GTG CAA AAT TAC GGC C-3′; reverse, 5′-AAA CGG CCG TAA TTT TGC ACG ATG C-3′). Oligonucleotide DNAs for the single-guide RNAs were annealed and inserted into lentiCRISPR v2 vector (LV-△N-IκBα-SV40-Puro) and digested with BsmBI restriction enzyme as described previously (41). All clones were confirmed by DNA sequencing using a primer 5′-GGACTATCATATGCTTACCG-3′ from the sequence of the U6 promoter that drives expression of sgRNAs. Cassettes of sgRNAs-Cas9 or pHR-CMV-LAMP3-IRES-puromycin were introduced to VK2 cells by using lentivirus-mediated gene transfer.

To investigate the role of the LAMP3 gene in HSV2 infection, LAMP3 expression was modified by gene knockdown and overexpression via a lentiviral vector. Lentiviruses were produced in HEK 293T cells using the standard calcium phosphate method (Life Technologies) as described previously (40). Briefly, 1 day before transfection, the cells were seeded into 6-well plates so that they would reach 70 to 80% confluence at the time of transfection. The cells were then transfected with lentiCRISPR v2 vector inserted with sgRNA or the lentiviral vector containing the LAMP3 transgene, the packaging plasmid psPAX2, and the envelope plasmid vesicular stomatitis virus G glycoprotein (VSV-G) using the calcium phosphate method (Life Technologies). After 18 h, the medium was replaced with growth medium supplemented with 10% FBS, and at 24 h after the medium change, lentiviruses were harvested in supernatants. The virus-containing medium was used to transduce VK2 cells. After 48 h of transduction, lentivirus-transduced cells were selected using antibiotic selection.

### Single-cell clone isolation.

The limiting dilution technique, as described previously (49), was used to isolate single-cell clones of both LAMP3 OE and LAMP3 KO cell lines. Transgenic cell lines were prepared individually in a homogenized cell solution to a concentration of 10 cells/mL. One hundred microliters of the diluted cell suspension was seeded into a 96-well plate and grown for 10 days in the presence of puromycin containing KSFM for selective selection of transfected cells. Wells with single cells were marked and allowed to grow for 10 to 15 days in the presence of puromycin (1 μg/mL; Invivogen, San Diego, CA, USA); the single-cell clone wells were then expanded subsequently.

### ALI culture and viral infection.

WT VK2 cells and the two transgenic cell lines were cultured in an ALI to mimic physiological conditions of the female genital tract as described (5). To generate ALI culture, a total of 60,000 cells from WT VK2, LAMP3 OE, or LAMP3 KO were seeded on 0.4-μm Transwell polystyrene inserts (BD Falcon, Mississauga, ON, Canada) with KSFM added to both apical and basolateral sides of the culture. The medium from the apical side was removed 24 h after seeding the cells, whereas the medium from the basolateral side was replenished every 24 h. After 7 days of culture, cells were infected with wild-type HSV-2 strain 333 at a multiplicity of infection (MOI) of 1 as described previously (5). The cells were then incubated at 37°C for 24 h, the supernatants were collected from the apical side, and the titer was determined by plaque assay on Vero cells as described previously (5). WT VK2, LAMP3 OE, and LAMP3 KO cell lines were also infected with HSV-2 tagged with green fluorescence protein (GFP) for 2 h and then washed and supplemented with fresh medium and incubated for a further 16 h. Cells were then visualized using an Evos FL digital inverted fluorescence microscope (Thermo Fisher Scientific, Burlington, ON, Canada) under a 20× objective. GFP-tagged HSV-2 derived from HSV-2 strain 333 with an EF-1 alpha promoter expressing GFP inserted between late genes UL26 (proteinase gene) and UL27 (gB gene) (kind gift of J. Vieira, University of Washington, Seattle, WA). The GFP expression was only visible at late stages of viral replication; therefore, at early stages of infection, immunofluorescence staining was performed to visualize virus.

### Immunofluorescent staining and confocal microscopy.

Immunofluorescent staining was performed as described previously (50). Briefly, cells were fixed in 4% paraformaldehyde and blocked for 30 min in blocking solution of 5% bovine serum albumin (MilliporeSigma, ON, Canada) and 5% normal goat serum (Millipore-Sigma) in 0.1% Triton X-100 (Bio-Rad Laboratories, ON, Canada) made in phosphate-buffered saline (PBS). The primary antibodies rabbit anti-EEA1, mouse anti-EEA1, rabbit anti-RAB7, rabbit anti-RAB11, and rabbit anti-LAMP1 (all from Abcam, Cambridge, UK), mouse anti-LAMP3 (Millipore-Sigma), and goat anti-HSV-2 (Abcam) were diluted in blocking solution in different combinations and incubated with cells for 1 h. Antibodies, the monolayers were washed with PBS and secondary antibodies including Alexa Fluor 488 goat anti rabbit IgG, Alexa Fluor 594 goat anti-mouse, and Cy5 donkey anti-goat IgG (Thermo Fisher Scientific) were added in two steps if all three secondary antibodies were used simultaneously. First, the Cy5 donkey anti-goat IgG was added and incubated for 1 h at room temperature. After extensive washing, Alexa Fluor 488 goat anti-rabbit IgG and Alexa Fluor 594 goat anti-mouse were added to cells and incubated for an hour, after which cells were washed, and Transwell cultures were excised from the polystyrene inserts and mounted on glass slides in Vectashield mounting medium (Vector Labs, CA, USA) with 4′,6-diamidino-2-phenylindole (DAPI). All samples were imaged on an inverted confocal laser-scanning microscope (Nikon Eclipse Ti2) using standard operating conditions (63× objective); optical laser thickness of 1 μm; image dimension of 512 × 512 pixels; and lasers, green, 488 nm; red, 594 nm; and Cy5, 633-nm or 647-nm laser lines. For each experiment, confocal microscope settings for image acquisition and processing were identical between control and treated cells, and three separate random images were acquired and analyzed for each experimental condition. Image quantitation analysis was done by Image J software (NIH, Bethesda, MD, USA) to measure the areas of both fluorescently stained specific protein and cellular nuclei. In the case of LAMP3 imaging, fluorescence and colocalization signals were only measured in cytoplasmic areas of cells to avoid any nonspecific fluorescence within the nucleus because LAMP3 is not known to enter the nucleus. Image J was used with the Coloc 2 plugin tool to create images showing colocalized points. The colocalized pixels in different fluorescence channels were calculated in 10 different images by the colocalization threshold plugin, and the obtained data were used in generating the correlation matrix using GraphPad Prism version 9.4.1. The significance between the correlation of colocalized fluorescence of two markers was analyzed by Pearson correlation analysis, and significance was determined by two-tailed *t* test.

### Endosomal/lysosomal inhibition analysis.

Cells were pretreated either with medium (untreated) or with the endocytosis inhibitor, Dynasore (Sigma-Aldrich, Oakville, ON, Canada) at a concentration of 80 μM for 1 h prior to HSV-2 infection. The cells were fixed at different time points for immunofluorescent staining of HSV-2, LAMP3, and the early endosomal marker 1 (EEA1). Confocal microscopy was performed as described earlier or previously described (50). For blocking endosomal trafficking and maturation into the late endosome, cells were pretreated with 20 μM EGA [4-bromobenzaldehyde-*N*-(2,6-dimethylphenyl), semicarbazone (*E*)-2-(4-bromobenzylidene)-*N*-(2,6-dimethylphenyl) hydrazinecarboxamide; MilliporeSigma (Calbiochem), Sigma-Aldrich] and infected with HSV-2 GFP at an MOI of 1. After 16 h, green fluorescent-infected cells were visualized under Evos FL digital inverted fluorescence microscope under a 20× objective. Cells grown in ALI cultures were also pretreated with EGA and infected with HSV-2 with an MOI of 1, and 24 h postinfection, supernatants were collected, and viral titration was performed using Vero plaque assay. For lysosomal inhibition, WT VK2, LAMP3 OE, and LAMP3 KO cell lines were pretreated for 24 h with a cocktail of inhibitors of the lysosomal pathway, including pepstatin A (29 mM), leupeptin (52 mM), and E-64 (69 mM) (all from MilliporeSigma) suspended in KSFM. The cells were pretreated with inhibitor cocktail and then infected with HSV-2 (MOI of 1), and the supernatants were collected from the apical side and their titers were determined by plaque assay on Vero cells as described (5).

### Viral titration.

Culture supernatants were collected for viral titration by Vero plaque assay as described previously (5). Briefly, for plaque assays, Vero cells (ATCC, Manassas, VA) were seeded a day before assay. Samples were diluted in serum-free α-MEM and added to monolayers. Infected monolayers were incubated at 37°C for 2 h with samples in serum-free medium to facilitate viral absorption and then overlaid with 5% serum containing α-MEM. Infection was allowed to occur for 48 h at 37°C. Monolayers were then fixed and stained with crystal violet, viral plaques were counted under a light microscope, and the number of PFU per milliliter was calculated by taking a plaque count for every sample and accounting for the dilution factor.

### Lactate dehydrogenase assay.

WT VK2, LAMP3 OE, and LAMP3 KO cells were grown in ALI culture. For the next 7 days, medium was added to the apical side for 1 h and then collected for lactate dehydrogenase (LDH) assay. Collected samples were used to measure cell stress by LDH assay kit according to the manufacturer’s instructions (Thermo Fisher Scientific). LDH in cell supernatant samples was compared with supernatant collected after complete lysis of cells by using the lysis buffer provided with the assay kit.

### Cell viability assay.

WT VK2, LAMP3 OE, and LAMP KO cells were seeded at a cell density of 10,000 cells/well in 24-well plates. Every 24 h, a set of wells was trypsinized, and the released cells were counted with a trypan blue exclusion assay. Cell growth and percent viability were graphed over time for 7 days.

### Quantitative reverse transcriptase PCR for measures of LAMP3 and HSV-2 gene expression.

To determine gene expression levels of LAMP1, LAMP2, LAMP3, and various HSV-2 genes, total RNA was extracted from HSV-2-infected cells at various time points postinfection using the RNAeasy kit (Qiagen, Toronto, ON, Canada) according to the manufacturer’s instructions and were subjected to cDNA synthesis using superscript cDNA synthesis kit (Invitrogen). Specific gene primers were used for amplification of each gene. LAMP3 primers were forward, 5′-GCGTCCCTGGCCGTAATT-3′, and reverse, 5′-TGCTTAGCTGGTTGCTGGA-3′. LAMP3 exon 1 primers used to confirm knockdown in LAMP3 KO cells were forward primer, 5′-GCC GTA ATT TTG CAC GAT -3′, and reverse primer, 5′-TGC TAA AGT TTG GTG AGG TGC T-3′. LAMP1 primers used were forward primer, 5′-GTT TCT TCA TTC TTT ACT G-3′, and reverse primer, 5′-TCT CTA CTG TTG TAA TGT-3′. LAMP2 primers used were forward primer, 5′-CGTTCTGGTCTGCCTAGTCC-3′, and reverse primer, 5′-CAGTGCCATGGTCTGAAATG-3′. The HSV-2-specific early gene primer used was immediate early gene ICP0, forward, 5′-GTGCATGAAGACCTGGATTCC-3′, and reverse, 5′-GGTCACGCCCACTATCAGGTA-3′. HSV-2-specific late gene primers used were VP16 forward, 5′-AATGTGGTTTAGCTCCCGCA-3′, and reverse, 5′-CCAGTTGGCGTGTCTGTTTC-3′, and gB forward, 5′-CGCATCAAGGACCACCTCCTC-3′, and reverse, 5′-GCTCGCACCACGCGA-3′. RT SYBR Green qPCR mastermix was used as per the manufacturer’s manual (Qiagen). RT-PCR was performed using StepOne Plus real-time PCR system (Thermo Fisher Scientific). After 10 minutes of a polymerase activation step at 95°C, 40 PCR cycles of 15 s at 95°C and 1 min at 60°C were performed. The fluorescence intensity was detected at the end of the annealing-extension step. Samples were run in duplicate, and all data were normalized to GAPDH (glyceraldehyde-3-phosphate dehydrogenase) mRNA expression as an internal control, while HSV-2 genes were compared with known HSV-2 standards. The specificity of amplification was confirmed by melting curve analysis. Relative gene expression was calculated using the threshold cycle (2^−ΔΔ^*^CT^*) formula and presented as fold change from mock-treated or untreated control cells.

### Statistical analysis.

The numeric data are reported as the mean ± standard error of the mean (SEM). GraphPad Prism version 9.4.1 (GraphPad Software, San Diego, CA, USA) was used to compare three or more means by one-way analysis of variance (ANOVA) for analyzing different treatments at the same time and two-way ANOVA for comparing two different variables with their specific controls. Student’s *t* test (unpaired) was used to compare two sets of data. Bonferroni posttest was performed to adjust the *P* value for multiple comparisons. *P* values for each analysis are indicated in figure legends. The level of statistical significance was defined at *P* values of <0.05.
